# Histological Stains in the Past, Present, and Future

**DOI:** 10.7759/cureus.18486

**Published:** 2021-10-04

**Authors:** Arslaan Javaeed, Shanza Qamar, Sundus Ali, Mir Ahmad Talha Mustafa, Areeba Nusrat, Sanniya Khan Ghauri

**Affiliations:** 1 Pathology, Poonch Medical College, Rawalakot, PAK; 2 Pathology, Rawalpindi Medical University, Rawalpindi, PAK; 3 Pathology, Ziauddin University, Karachi, PAK; 4 Emergency Medicine, Shifa International Hospital, Islamabad, PAK

**Keywords:** microtomes, ziehl-neelsen stain, histology, histopathology, histological stains, histochemistry, immunohistochemistry, surgical pathology

## Abstract

Certain contemporary histology stains and methods are not the same as those used in the past. This progression has delved into the requirement for more precise, less complex, and efficient staining procedures. The objective of this study is to assess historical and contemporary stains and procedures, as well as the challenges surrounding their improvement. Carmine, hematoxylin, silver nitrate, Giemsa, trichome stain, Gram stain, and mauveine were among the first histological stains discovered in nature. Aside from their utility in the study of tissues at the time, they also laid the groundwork for the development of commercial dyes that are still in use today. Hematoxylin and eosin, Ziehl-Nielsen (ZN) stain, periodic acid-Schiff stain, and Grocott-Gomori methenamine silver stain are some of the most recently developed histological stains. The future of histological stains and processes appears to be influenced by technological advancements and the demand for cost-effective diagnostic approaches in the healthcare system. Thus, currently used histological stains appear to be economical, quick, and reliable tools for interpreting, archiving, and delivering essential diagnoses that could not be achieved by any other means.

## Introduction and background

The practice of histology refers to the microscopic study of plant and animal cells and tissues through the processes of staining, sectioning, and studying them under either a light or electron microscope [1]. There are numerous approaches applied to the assessment of the microscopic components of the cells and other tissue characteristics, some of which have been employed in an autopsy, forensic investigations, and diagnosis [1].

Process of histological staining

The process of histological staining involves five primary stages, namely fixation, processing, embedding, sectioning, and staining.

Fixation

Fixation is the addition of special substances such as chemicals to tissues under investigation to preserve them by halting the progression of various biochemical processes that lead to degradation [1]. Some of the fixatives commonly used include formalin, neutral buffered formalin (NBF), and glutaraldehyde. Fixation has been shown to alter the structure of the nucleus, and thus genetic material cannot be studied upon fixation. This has led to Bouin's fixative for soft and delicate tissues such as those obtained from the brain or an embryo. Bouin fixative serves as an ideal preservative for glycogen and nuclei, but it is also known to distort the structure of kidney tissues and the mitochondria [2].

Paraffin embedding

Due to the hydrophobic nature of the wax used during fixation, clearing the tissue of any water is crucial to a perfect waxing step and also serves to harden the tissues in preparation for sectioning. This is done during dehydration which involves putting the tissue through a series of alcohol-water solutions. Further to this, the replacement of the alcohol with histoclear or cedar oil prepares for the introduction of wax into the tissue specimen. Finally, the tissue is inserted into fresh paraffin for ample time to let it cool.

Sectioning

Sectioning is the process of obtaining microtomes (small tissues sections) that allow light to pass through since they are viewed under microscopes. It involves cutting paraffin-embedded or frozen tissue into thin translucent slices creating a single plane of focus. The slices are then mounted into a slide for investigation using a microscope. To preserve the tissues, mounting it into a glass slide using transparent substances to harden and covering it with a thin glass slip that seals the preparation is critical in ensuring the readiness of the tissue for use. Sectioning ensures that the tissues under investigation are clear and provide desired results and details. Significantly, serial sections give room for the 3D structure of tissue to be visible [1]. This consideration is vital in determining the abnormality of tissue under investigation. 

Staining

The addition of a dye to highlight abnormalities and improve the contrast between tissues is referred to as staining [3-6]. Hematoxylin, for example, colours the nucleus blue after being administered, whereas eosin stains it pink, producing contrast in tissues that absorb either dye. As a result, histological staining is a multistep procedure that involves a variety of stains and other chemicals that may interact with other compounds found in tissues to change the results [3-6]. We glanced at the changes that have occurred in the histological staining process and advancement, as well as the likely reasons for these alterations. The changes were made primarily to make histological staining easier, faster, cheaper, and more accurate.

## Review

Methods

A literature search on online medical databases for scholarly articles published from June 2011 to June 2021 was carried out on PubMed, National Center for Biotechnology Information (NCBI), Cumulative Index to Nursing and Allied Health (CINAHL) Medscape, EBSCO, Medline, and PsycINFO using keywords such as ‘histochemistry’, ‘histopathology’, ‘immunohistochemistry’, ‘histology’, and ‘surgical pathology’. A total of 7,519 peer-reviewed articles were generated. The inclusion criteria required journals that discussed the use of histological stains used in the past, as well as papers touching on the advancements of technology. Studies excluded from the review included those that were not initially published in the English language and those focussing on plants. Additionally, 479 articles were excluded since they demonstrated characteristics similar to pseudo-journals leaving a total of 48 scholarly papers that met the criteria for inclusion in this review. 

Results

Histological Stains From the Inception

Staining specimens was a science that followed the discovery of the light microscope by Antony van Leeuwenhoek in 1747 [7]. Ancient staining techniques mainly revolved around this development since the crude instruments required simple dyes that existed naturally. These dyes included madder, indigo, and saffron, among others. Specimen fixation was also simply done using readily available chemicals such as potassium dichromate, alcohol, and mercuric chloride [8]. There were many staining procedures available at the time, and they were chosen based on the type of tissue being studied. Carmine, a brilliant crimson pigment originating from cochineal insects found in Mexico, is one of the oldest stains in use. John Hill, a scientist interested in the histology study of wood, first introduced this dye in 1770 [9]. The Prussian blue dye was first used in 1774 to aid in the histochemical identification of hemosiderin in tissues. [10].

Alturkistani, Tashkandi, and Mohammed Saleh carried out a study that discussed the discovery of a purple dye by William Henry Perkin, which was later named mauveine. This dye provided the fundamentals for the aniline family of dyes. Accordingly, scientists such as Ehrlich keenly studied the aniline class of dyes and were able to successfully identify the different types of white blood cells using methylene blue as the stain [1]. Further to this, Joseph von Erlach, the father of microscopy, introduced newer techniques of processing specimens and led to the development of carmine to stain cerebellum cells in 1818 [11,12]. The aniline dye was later exhaustively described by German scientists [13].

Meanwhile, Microbiologists Alice B. Schaeffer and MacDonald Fulton discovered the endospore staining [14]. This invention was used to identify the presence or absence of endospores which was postulated to be the main cause of difficulties in the treatment of bacterial diseases caused by spore-forming bacterial organisms such as *Bacilli anthracis* [14]. Two distinct methods are used to perform staining in endospore staining. The Schaeffer Fulton stain uses malachite green dye, and Safranin to conduct staining. On the other hand, the Dorner method uses carbolfuchsin stain, acid alcohol, and Nigrosin solution [14]. Most bacterial spores are difficult to stain. Therefore, this discovery is useful in eliminating bacteria causing diseases in humans, which could potentially lead to adverse health degradation. 

Immediate and Potential Strategies' Emergence

The growth of scientific knowledge increased the breadth of diagnostic diseases, and the discovery of more complex microscopes led to the development of more complex stains that were superior to their predecessors' in tissue visualization capabilities. According to previous studies, it all began with the inauguration of double staining techniques in the 1870s, which led to the formulation of the hematoxylin and eosin (H&E) stain. Hematin and hematoxylin refer to substances that occur naturally and have been incorporated as agents of histopathology. Wilhelm von Waldeyer developed the hematin stain from readily available logs of trees found in Central America. Hematoxylin was an enhanced version of the dye due to its weak nature [15]. 

Following the discovery of disease-causing bacteria, Hans Christian Gram developed the Gram staining technique for microbe identification. Crystal violet, Gram's iodine, and safranin are some of the stains used in this procedure. To distinguish Gram-positive from Gram-negative bacteria, the approach incorporates concepts of alcohol decolorization and oil immersion [16-18]. Following thereafter, procedures like the Ziehl-Neelsen strain were discovered that were acid-fast. Because anile oil may penetrate the tubercle bacillus, the mycobacterium that causes tuberculosis, German bacteriologist Franz Ziehl and pathologist Friedrich Nielsen devised this approach [19].

Advancements in histological tissue processing led to the development of a quicker diagnostic method; frozen sections. This technique featured the use of paraffin infiltration into the tissues. The frozen tissue section technique is a quick and accurate tissue study technique. During the removal of tumors, surgeons may await results of masses sampled intraoperatively to make a diagnosis and a quick decision on the extent of resection needed during the surgery [20,21].

Immunoperoxidase and immunofluorescence, which have been created to provide descriptions of the genetic make-up of individual cells, are in the future of histological stains. Unlike their predecessors, who destroyed nuclear material, these breakthroughs enable the study of nuclear material [22].

Discussion

The genesis of classical staining techniques may be traced back to the works of scientist Leeuwenhoek whose microscopes could only use simple stains. He was known for using stains such as indigo, saffron, and Madder to stain tissues for use with his crude instruments. This era was marked by simplicity as stains were naturally available and simple to extract. The microscopes at the time were crude instruments that did not require the synthesis of complex dyes. Similarly, the essence of microscopy was simple; to determine the characteristics of various cells, their similarities and differences more so in comparison with plant cells. Joseph Von Gerlach, the pioneer of microscopy staining, successfully used ammoniacal carmine in his technique which was mainly focussed on further characterization of cells. He specifically studied cells of the cerebellum which had not been studied previously. The early histologists are known to have also used readily available chemicals to prepare tissues for study under the microscope. These chemicals include alcohol, mercuric chloride, and potassium dichromate, all of which were used to harden cellular tissues [7,8,12].

The expanding breadth of medical knowledge and emerging complex medical conditions led to the need for further characterization of human tissues. The study of specimens under a microscope had proved to be a resourceful scientific tool. Further discoveries led to the development of colored staining agents such as silicone and trichrome to improve the contrast between various tissues. The colored stains are still applied today in the case of renal and liver biopsies [23].

Other discoveries that altered histological practices include the postulation of the germ theory of disease, stating that every disease had a disease-causing organism. This necessitated the development of staining techniques that could identify these microorganisms and differentiate between Gram-positive cells and Gram-negative microorganisms by nature of their cellular characteristics and stain absorption properties. Gram stain technique applies the use of stains such as crystal violet as an initial stain, Gram's iodine, and safranin or fuschin as a counterstain. The technique also applies principles of alcohol decolorization and oil immersion to help differentiate Gram-positive from Gram-negative bacteria. In Gram-positive cells, there is the uptake of the initial stain which is incapable of decolorization, whereas the Gram-negative cells are decolorized and take up the color of the counterstain as an identifier. Though the stains are still used to date, they risk being obsolete following the development of better stains and diagnostic methods [24-26].

There was a need to enhance the science of histology and the stains to utilise as other creatures other than bacteria were discovered. One such organism is the Mycobacterium group of organisms that have a high lipid content. Thus, they could not take up any of the previously discussed stains. The spread and declaration of tuberculosis as a pandemic led to the development of acid-fast staining techniques such as the Ziehl-Neelsen stain. The technique relies on the fact that anile oil can penetrate the tubercle bacillus, the mycobacterium which causes Tuberculosis and other similar organisms. Closely related techniques that were developed include Auramine/Rhodamine staining procedure that has shown diagnostic superiority in comparison to its predecessor [27]. Despite the fact that these two staining procedures and their respective stains have been criticised for their failure to provide information on disease-resistant strains, they are nevertheless employed to study microorganisms today. This is due to the fact that there are no better or less expensive stains or staining processes available. Furthermore, they add to the findings of other diagnostic research such as molecular or culture studies [28]. 

Hematoxylin, which stains the nucleus of the cell blue, and eosin, which stains the connective tissue, cytoplasm, and other extracellular characteristics red, are two other early stains that are still widely employed in light microscopy. At its discovery, it was viewed as a seemingly weak stain that had to be combined with other solutions while in its oxidized state. It was discovered that when used together with an oxidizer mordant, it has a heightened ability to differentiate cells and also provide resistance to acidic solutions that may be present in cells under study hence it has stood the test of time to ensure its use in current practice [15,29].

Silver nitrates were traditionally used to improve the visibility of tissue structures. The use of the dye diminished with time since confirmatory tests were required when silver nitrate is applied. Also, the efficiency of the silver nitrate stain is diminished by argentaffin cells which are located in the intestines, lungs, and melanin. Nevertheless, scientists have developed a means of curtailing the occurrences of argyrophilic reactions if silver nitrate is used in the staining process. Most notably, Grocott-Gomori and Gomori reticulin approaches were formulated to evaluate the diseases and missing tissues in the rectum and liver. The Romanowsky-Giemsa stains are multi-colored and are therefore used to identify blood parasites. The stain has been improved to make it suitable for formalin-fixed, bone marrow, and paraffin-embedded biopsies [30-32].

Additionally, the endospore staining techniques designed by microbiologists Alice and MacDonald made a huge impact on the elimination of bacteria within the human body. Endospore staining is one such critical invention that has played a major role in identifying endospore-forming bacterial pathogens [33,34]. For instance, endospore staining led to the identification of *Clostridium difficile* pathogen. This bacterium is the cause of severe diarrhea among millions of individuals in the world. Because of its medical importance, a more enhanced and exact procedure was developed to assure proper staining [35]. One such method is the Wirtz method which involves using heat fixation and counterstain. This method has been proved to provide exceptional results upon examination by identifying endospore-forming bacteria [36]. The endospore-forming bacteria will be stained green while the rest will appear red. Importantly, endospore staining is useful in the detection of the firmicute group of bacteria such as the Bacillus spp. This type of bacteria is known to cause infections that are related to trauma and systemic infections. Therefore, this discovery is critical in the public health arena due to its usefulness to treat diseases such as meningitis which could potentially result in death to individuals. This staining is also used in the differentiation of spore-producing bacteria from other forms such as vegetative bacteria [36,37].

Recent advancements in medical practice have resulted in the existence of multiple experts in diagnostic techniques and massive discoveries of histological methods that are fast, safe, and of higher specificity and sensitivity in the detection of diseases. Such tests include intraoperative staining techniques such as frozen section studies. This technique employs the modification of hematoxylin and eosin stains but using a quicker method to obtain results. The flexibility of these techniques and stains used today to allow for the use of these stains in the future as they have incorporated well with the advancement in technology such as the automatic analysis of segments, further analysis, their interpretation, and recording for future reference [38,39]. 

Furthermore, immunohistochemistry (IHC), which provides a key framework for diagnostic pathology, is projected to dominate existing and future staining. Immunohistochemistry is a technique that uses the notion of antigen binding to an antibody to diagnose a disease by identifying specific proteins. Immunoperoxidase and immunofluorescence antibodies, which primarily highlight proteins in cells, are the most often used stains in IHC. Immunoperoxidase is used in diagnostic surgical pathology to provide additional information that may not be identified quickly using hematoxylin and eosin stains. The fact that molecules are recognised in situ in the cell structure is an extra benefit. IHC is commonly used in modern surgical pathology to determine cancer cell types, as well as the different subtype classifications that may occur, and the likely cell of origin, especially in metastatic cancer in primary sites where the cell of origin may be unclear [40,41]. 

Another change that is anticipated in the future of histology is a revamp in surgical pathology reports to include microscopic and gross photographs of specimens that have been digitally processed. Moreover, reports on tumor specimens may consist of DNA analysis, cytometry, proteomic, and Automated Cellular Imaging Systems. The role of the pathologists is also expected to expand to include the consolidation of numerous laboratory reports from different clinicians to generate a comprehensive document. Such a report which predominantly focuses on molecular pathology would be instrumental in informing on the ideal chemotherapy or treatment intervention that is to be applied to a specific patient to ensure maximum effect [42,43]. 

In addition to that, there is an increased desire to ensure faster turnaround time (TATs) owing to the surge in healthcare costs. Since patients are compelled to occupy expensive hospital beds, physicians would want to provide a reduction in the length of stay in the hospital hence the urgency in releasing pathology results on the same day to facilitate the immediate commencement of treatment or discharge. Accordingly, the requirement for cost-effectiveness is to ensure patients are sent home with the right diagnosis and treatment plan. The advancement in technology has contributed to the development of computer programs that facilitate pattern recognition which is used to determine changes in microscopic tissue structure. In essence, researchers have discovered mechanisms of using computerized image analysis to assign scores to progesterone-receptor and estrogen-receptor biomarkers, particularly for invasive ductal carcinoma. Equally important is the fact that there have been technological advancements in regards to the creation of tissue processors. In contemporary histological practice, these processes use thin slices of tissues which are processed in approximately one hour [42,44]. 

Other newer stains, such as the revolutionary tetra chrome procedure, which uses Alcian Blue, Periodic Acid - Schiff's, Haematoxylin, and Picric Acid stains after fixation of the tissues with Canoy's solution and embedding in paraffin, are also being investigated. The technique provides excellent images that can be incorporated into today’s high-resolution digital screens. Lastly, since many of the current histological studies focus on tumor diagnosis due to the latest emphasis on early diagnosis and treatment of cancers, there has been extensive research on the extent of immunohistochemistry application. Numerous findings are expected on the use of antibodies and IHC in conjunction with algorithms to predict which tumors are likely to express specific antibodies [45,46].

## Conclusions

In summation, histopathological stains have improved over time, resulting in more precise, quicker, more productive, safer, and less difficult dyes and procedures. It is also worth noting that ancient stains have not been completely abandoned, but rather have been modified by their flaws. This helps to explain why old stains are still used today. For a cost-effective healthcare system, modern medicine has stressed prompt diagnosis, early management, and a subsequent shorter hospital stay. The incorporation of technology in medicine has not spared histology either, and new stains such as Alcian Blue, Periodic Acid-Schiff's, Haematoxylin, and Picric Acid stains have been devised to use histological techniques that allow for automatic processing, interpretation, and digital recording of results for future reference resulting in more accurate and quicker histological methods.
